# Comparison of Clinical Outcomes Between Antegrade and Retrograde Techniques for Chronic Total Occlusion Revascularizations: A Systematic Review and Meta-Analysis

**DOI:** 10.7759/cureus.66213

**Published:** 2024-08-05

**Authors:** Godfrey Tabowei, Monica Kontor, Mandeep Kaur, Revanth Reddy Bandaru, Sara Ahmed, Komal Kumari, Calvin R Wei, Neelum Ali

**Affiliations:** 1 Internal Medicine, Texas Tech University Health Sciences Center, Odessa, USA; 2 Internal Medicine, Korle Bu Teaching Hospital, Accra, GHA; 3 Internal Medicine, HCA Capital Regional Medical Center, Tallahassee, USA; 4 Internal Medicine, East Carolina University, Greenville, USA; 5 Emergency Department, National Institute of Cardiovascular Diseases, Karachi, PAK; 6 Internal Medicine, Ziauddin University, Karachi, PAK; 7 Research and Development, Shing Huei Group, Taipei, TWN; 8 Internal Medicine, University of Health Sciences, Lahore, PAK

**Keywords:** systematic review and meta analysis, pci, chronic total occlusion, antegrade, retrograde

## Abstract

Chronic total occlusions (CTOs) present significant challenges in interventional cardiology. This meta-analysis aims to compare the efficacy and safety of retrograde versus antegrade techniques in CTO percutaneous coronary intervention (PCI). A systematic review and meta-analysis were conducted following PRISMA guidelines. Electronic databases were searched through June 20, 2024. Studies comparing outcomes between antegrade and retrograde methods for CTO-PCI were included. Primary outcomes were procedural and technical success. Secondary outcomes included major adverse cardiac events (MACE), all-cause mortality, and myocardial infarction. The final analysis included seventeen studies. The antegrade approach showed a 5% higher likelihood of technical success (OR: 1.05, 95% CI: 1.02-1.09) and 14% higher odds of procedural success (OR: 1.14, 95% CI: 1.10-1.19) compared to the retrograde approach. The antegrade group also demonstrated lower risks of MACE, all-cause mortality, and myocardial infarction (RR: 0.40, 95% CI: 0.26-0.63). This meta-analysis suggests that the antegrade approach in CTO-PCI is associated with higher success rates and lower risks of adverse outcomes compared to the retrograde approach. However, the retrograde technique remains crucial for complex lesions and patients with multiple comorbidities.

## Introduction and background

Chronic total occlusions (CTOs) represent a significant challenge in interventional cardiology, defined as coronary artery lesions with thrombolysis in myocardial infarction (TIMI) flow grade 0 for at least three months [1]. These complex lesions, characterized by dense fibrocalcific plaque, microvascular channels, negative vessel remodeling, and often ambiguous proximal and distal caps, are found in approximately 15-30% of patients undergoing coronary angiography [2]. The presence of CTOs is associated with an increased risk of adverse cardiovascular events, reduced left ventricular function, and higher mortality rates [3]. Although numerous retrospective studies have demonstrated the prognostic advantages of successful CTO revascularization, there remains no consensus on the clinical indications for its treatment [4-5]. Additionally, treating CTOs with percutaneous coronary intervention (PCI) poses more significant technical difficulties compared to non-occlusive lesions. This is characterized by lower procedural success rates, a higher likelihood of complications during the procedure, and increased rates of restenosis compared to PCI for non-CTO lesions [6].

The transformation of CTO-PCI success rates from less than 70% to about 90% was made possible by the development of retrograde CTO crossing procedures. The retrograde technique has been linked, albeit not exclusively, to longer procedural times, higher contrast and fluoroscopy usage, and an increased risk of periprocedural and perhaps long-term adverse cardiac events [7]. In contrast, the antegrade approach remains the primary and initial strategy for most CTO interventions. It typically involves fewer complex techniques and is generally associated with shorter procedure times and lower resource utilization [8]. The antegrade method has also evolved significantly, with the development of specialized techniques such as antegrade wire escalation (AWE) and antegrade dissection and re-entry (ADR). These advancements have improved the efficacy of the antegrade approach, allowing for the successful crossing of increasingly complex lesions [9]. 

The choice between these techniques often depends on various factors, including lesion characteristics, collateral circulation, and operator experience. However, the current literature presents conflicting evidence regarding the superiority of one technique over another in terms of procedural success, safety, and long-term outcomes. Given this context, there is a clear need for a comprehensive synthesis of the available evidence to guide clinical decision-making and optimize patient outcomes. This meta-analysis aims to systematically compare the efficacy and safety of retrograde versus antegrade techniques in CTO-PCI.

## Review

Methodology 

The Preferred Reporting Items for Systematic Review and Meta-Analysis (PRISMA) statement's guidelines were followed in conducting the present systematic review and meta-analysis.

Literature Search 

We conducted a systematic and thorough electronic search from the inception of databases to June 20, 2024, in Embase, PubMed, and Web of Science without restriction to location or language of publication. The following was a list of search terms used in the literature search: “chronic total occlusion,” “retrograde,” “revascularization,” and “antegrade” with medical subject heading (MeSH) terms. These terms were combined together using boolean operators (“AND,” “OR). To find any other potentially relevant papers, a manual review of the bibliographies of relevant and included studies was also carried out.

Eligibility Criteria 

We included published research comparing outcomes between antegrade and retrograde methods for CTO-PCI. We included the most recent or thorough publication if multiple studies reported the same patient cohort's outcomes. For research that presented outcomes at various intervals, we used outcomes at maximum follow-up. The initial search was conducted separately by two investigators, who then vetted titles and abstracts, removed duplicate records, and eliminated extraneous literature. Next, in full-text format, the remaining possibly relevant literature was evaluated to establish eligibility. Disagreements or conflicts were settled through consensus.

Data Extraction 

Using a standardized data extraction form, one author extracted pertinent information from the study's included publications, and two other authors independently confirmed the information's accuracy. The information gathered included several components, including the principal author, the year of publication, the region where the study was conducted, the study design, the follow-up duration, the sample size, and the outcome data. Collaborative talks with coauthors addressed data validation and discrepancies.

*Study Outcomes* 

The primary outcomes assessed in this study included procedural success and technical success. Technical success was defined as achieving TIMI 3 blood flow with less than 30% residual stenosis. Procedural success required this technical success to be achieved without any major adverse cardiac events (MACE) occurring during the hospital stay. Other endpoints included MACE, all-cause mortality, myocardial infarction, and revascularization.

Statistical Analysis 

Statistical analysis was performed using Review Manager Software 5.4.1 (The Nordic Cochrane Centre, The Cochrane Collaboration, Copenhagen, Denmark). Odds ratios (ORs) with 95% confidence intervals (CIs) were presented as summary statistics for the outcome variables. A statistically significant result was determined by a p-value less than 0.05. Statistical heterogeneity was evaluated using I2 statistics, where an I2 value greater than 50% was deemed substantial and an I2 value exceeding 75% was considered considerable. Due to expected significant clinical and methodological heterogeneity, we utilized the random-effects generic inverse variance method to calculate the OR.

Results

**Figure 1:**
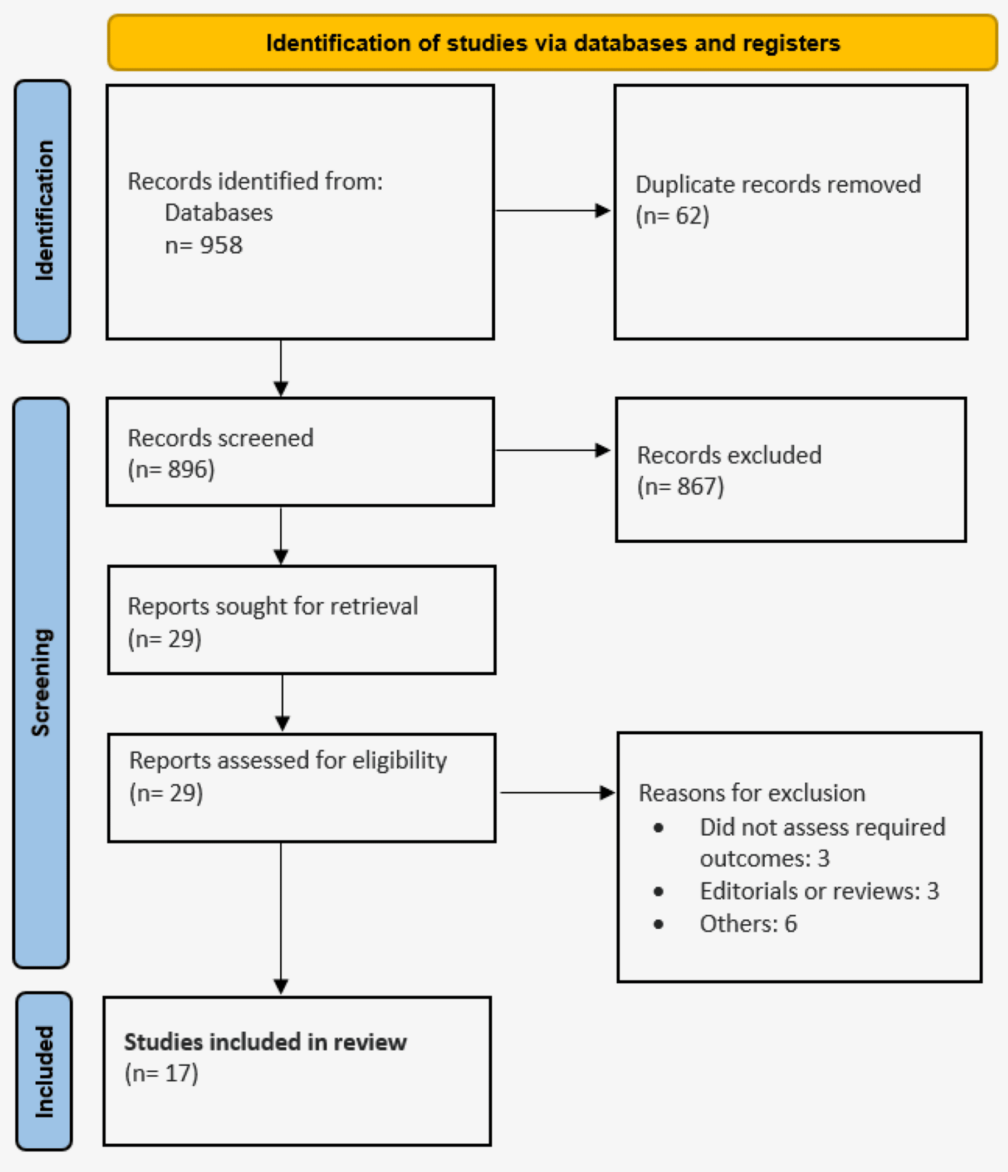
PRISMA flowchart of study selection

The process of identifying, screening, and selecting relevant studies is shown in the PRISMA statement flowchart (Figure 1). From the original electronic database search, we found 958 citations. After deleting duplicates and going over the titles and abstracts, 29 articles were thought to be appropriate records for inclusion. Following a thorough full-text review, the meta-analysis eventually contained 17 papers. The primary attributes of the included studies are outlined in Table 1, encompassing studies conducted in Egypt, Germany, the United States, Japan, Korea, Taiwan, and China. Four studies enrolled participants from multiple countries. These studies were published between 2011 and 2024.

**Table 1:** Study characteristics NR: not reported

Author name	Year	Region	Design	Follow-up	Groups	Sample size	Age	Male	Hypertension	Diabetes	Multivessel disease
Bendary et al. [10]	2022	Egypt	Prospective	In hospital	Antegrade	75	NR	NR	NR	NR	NR
					Retrograde	77					
Bijuklic et al. [11]	2018	Germany	Retrospective	24 months	Antegrade	325	63.7	280	322	87	197
					Retrograde	71	61.8	62	70	13	34
Elmaksoud [12]	2023	Egypt	Retrospective	6 months	Antegrade	30	62.03	25	18	19	NR
					Retrograde	30	60.03	24	18	17	
Etriby et al. [13]	2024	Egypt	Prospective	6 months	Antegrade	62	NR	NR	NR	NR	NR
					Retrograde	18					
Galassi et al. [6]	2011	Multinational	Retrospective	In hospital	Antegrade	1749	NR	NR	NR	NR	NR
					Retrograde	234					
Kalra et al. [14]	2020	Multinational	Retrospective	12 months	Antegrade	431	65.1	342	363	157	NR
					Retrograde	454	65.7	362	400	206	
Karmpaliotis et al. [15]	2016	United States	Retrospective	In hospital	Antegrade	745	65.1	609	669	343	NR
					Retrograde	531	66.1	465	474	231	
Kwon et al. [16]	2014	Korea	Retrospective	56.4 months	Antegrade	908	NR	NR	NR	NR	NR
					Retrograde	243					
Lee et al. [17]	2017	Taiwan	Retrospective	In hospital	Antegrade	152	65.6	122	135	52	NR
					Retrograde	169	61.8	155	138	51	
Michael et al. [18]	2014	United States	Retrospective	24 months	Antegrade	152	63.6	151	132	64	NR
					Retrograde	41	63.7	39	36	17	
Suzuki et al. [19]	2017	Japan	Retrospective	In hospital	Antegrade	1872	66.8	1593	1460	841	243
					Retrograde	724	66.9	639	584	332	120
Tajti et al. [20]	2020	Multinational	Retrospective	In hospital	Antegrade	2603	64.2	2171	2361	1093	NR
					Retrograde	1505	65	1290	1361	637	
Tanaka et al. [21]	2018	Japan	Retrospective	84 months	Antegrade	507	67.4	435	412	226	208
					Retrograde	248	64.7	259	232	110	126
Werner et al. [22]	2014	Germany	Retrospective	In hospital	Antegrade	386	64.9	324	300	104	321
					Retrograde	106	62.2	91	91	27	88
Wu et al. [23]	2020	Multinational	Retrospective	In hospital	Antegrade	259	62.2	229	189	90	153
					Retrograde	226	60.6	198	164	76	158
Wu et al. [24]	2023	China	Retrospective	In hospital	Antegrade	25	63.36	17	13	6	8
					Retrograde	64	65.22	38	39	19	19
Zivelonghi et al. [25]	2018	Netherlands	Retrospective	12 months	Antegrade	285	62.2	224	162	52	NR
					Retrograde	45	64.9	35	22	13	

Outcomes 

**Figure 2:**
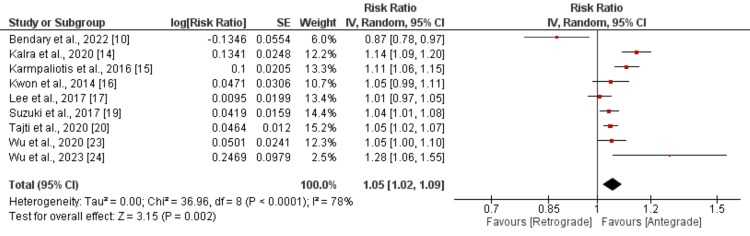
Comparison of technical success between retrograde and antegrade approach Sources: References [10,14-17,19-20,23-24]

**Figure 3:**
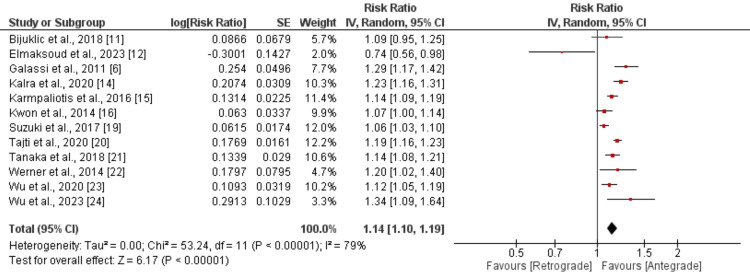
Comparison of procedural success between retrograde and antegrade approach Sources: References [6,11-16,19-24]

Technical and procedural success: Nine studies compared the technical success rates, revealing that patients undergoing the antegrade approach had a 5% higher likelihood of technical success than those using the retrograde approach (OR: 1.05, 95% CI: 1.02 to 1.09). This difference was statistically significant (p-value: 0.005), as illustrated in Figure 2. Significant heterogeneity was observed among the study results (I²: 78%). The pooled analysis results for procedural success between the two groups are shown in Figure 3. This analysis indicated that the odds of procedural success were significantly higher for patients with the antegrade approach compared to the retrograde approach (OR: 1.14, 95% CI: 1.10 to 1.19), with notable heterogeneity among the study results (I²: 79%).

**Figure 4:**
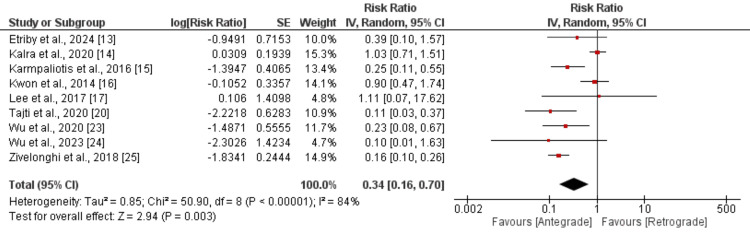
Comparison of MACE between antegrade and retrograde approach Sources: References [13-17, 20, 23-25]

MACE: Nine studies in this meta-analysis evaluated MACE, with the results presented in Figure 4. The pooled analysis indicated a lower risk of MACE in the antegrade group compared to the retrograde group (RR: 0.34, 95% CI: 0.16 to 0.70). The study results showed high heterogeneity (I²: 84%). A sensitivity analysis, including five studies only that assessed in-hospital outcomes, revealed similar results to the pooled analysis but with reduced heterogeneity from 86% to 0% (RR: 0.21, 95% CI: 0.12 to 0.36). This showed that the duration of follow-up is one of the reasons for heterogeneity in the pooled analysis.

**Figure 5:**
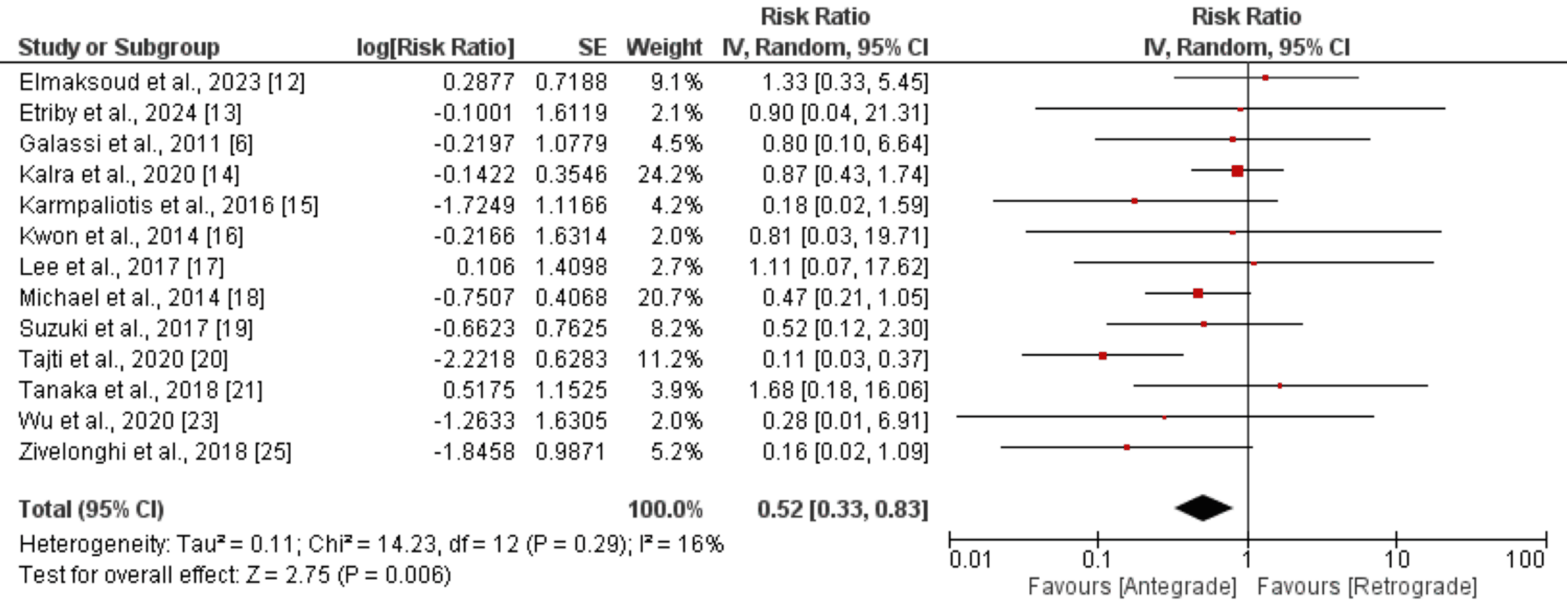
Comparison of death between antegrade and retrograde approach Sources: References [6,12-21,23,25]

All-cause mortality: We included 14 studies to compare the risk of all-cause mortality between two groups, and the results are depicted in Figure 5. We found that the risk of all-cause mortality was significantly higher in patients in the antegrade group compared to the retrograde group (RR: 0.52, 95% CI: 0.33 to 0.83). No significant heterogeneity was reported among the study results (16%). We performed subgroup analysis based on the duration of follow-up. For long-term follow-up (follow-up of 1 year or more), we found lower mortality risk in the antegrade group (RR: 0.64, 95% CI: 0.39 to 1.04) in a pooled analysis of five, but the difference was insignificant (p-value: 0.07) and the I-square value showed no heterogeneity (I-square: 1%). Studies assessing in-hospital death (seven studies) also showed lower risk in patients in the antegrade group, but the difference was significant (RR: 0.28, 95% CI: 0.13 to 0.58). No heterogeneity was reported among the study results (I-square: 0%).

**Figure 6:**
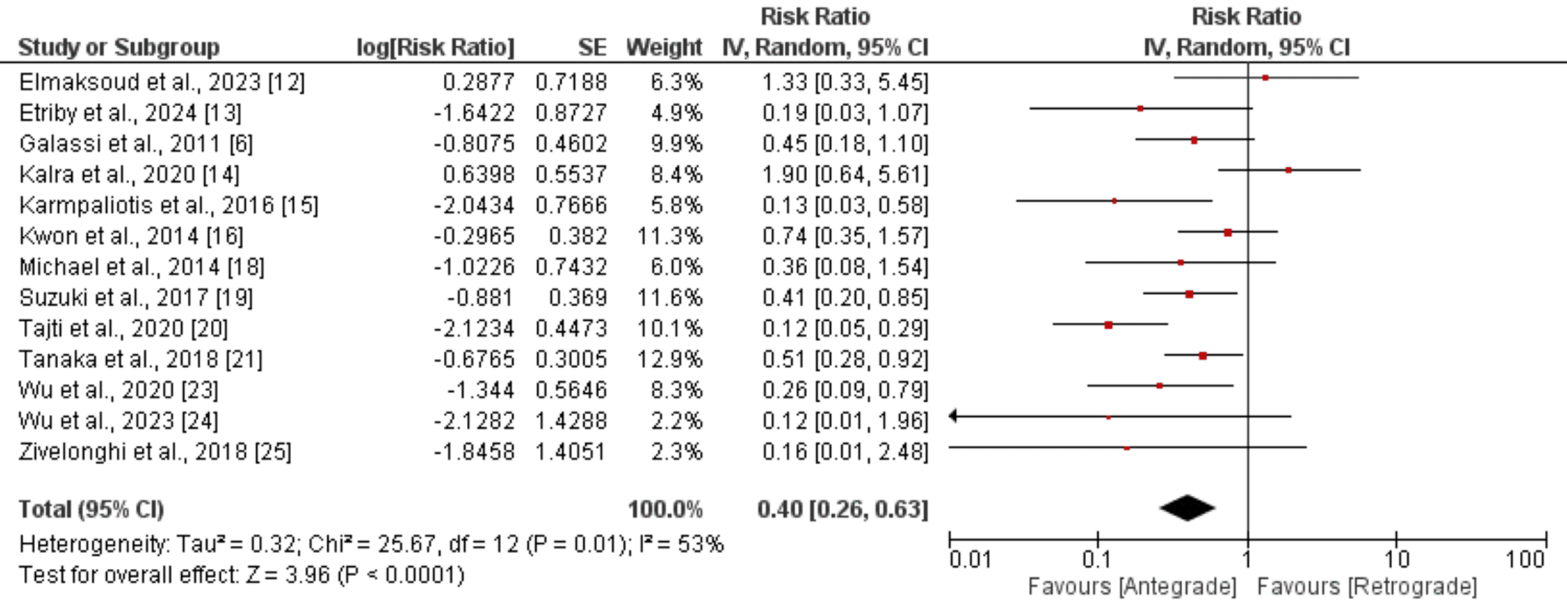
Comparison of myocardial infarction between antegrade and retrograde Sources: References [6,12-16,18-21,23-25]

Myocardial infarction: The pooled analysis of comparing the risk of myocardial infarction included 12 studies, and the results are presented in Figure 6. Pooled analysis showed a higher risk of myocardial infarction in the retrograde group compared to the antegrade group (RR: 0.40, 95% CI: 0.26 to 0.63). Significant heterogeneity was reported in the study (I-square: 53%). We performed subgroup analysis based on the duration of follow-up. For long-term follow-up (follow-up of one year or more), we found lower myocardial infarction risk in the antegrade group (RR: 0.67, 95% CI: 0.38 to 1.17), but the difference was insignificant (p-value: 0.16) and the I-square value showed no significant heterogeneity (I-square: 35%). Studies assessing the in-hospital risk of myocardial infarction also showed lower risk in patients in the antegrade group, but the difference was significant (RR: 0.34, 95% CI: 0.22 to 0.53). No heterogeneity was reported among the study results (I-square: 0%).

Discussion

The present meta-analysis compared outcomes between antegrade and retrograde crossing techniques in CTO-PCI. The results of this meta-analysis showed that the technical and procedural success were significant better in patients underwent antegrade CTO-PCI compared to their counterparts. Additionally, risk of MACE, all-cause mortality and myocardial infarction were also significantly lower in patients underwent antegrade CTO-PCI compared to retrograde CTO-PCI. After analyzing 12 observational studies with 10,240 patients, Magaly et al. discovered that CTO lesions treated with an antegrade-only strategy had a greater success rate and a lower J-CTO score [26]. 

According to our research, the retrograde method increases the risk of myocardial infarction because of the prolonged occlusion of collateral channels by the microcatheters and retrograde wire [26], which has been linked to worse long-term outcomes and increased mortality. However, an antegrade-only strategy would not have been able to revascularize the majority of the CTOs included, and some of the issues linked to the retrograde approach would have arisen during antegrade crossing attempts. Furthermore, patients from the early 2000s were included in some of the studies in our study, which was at an early stage of the retrograde procedures' learning curve. A higher risk of restenosis is linked to the implantation of longer, smaller-caliber stents, which is typically the result of retrograde PCI in more difficult anatomical settings [27]. Furthermore, the retrograde approach's common utilization of dissection and re-entry procedures may raise the rate of restenosis [28-29]. 

Retrograde CTO-PCI procedures have become less used in modern practice due to concerns about the rate of problems associated with them. The usage of retrograde procedures decreased statistically significantly from 39% in 2012-2016 to 29% in 2017 to 2019, according to data from the PROGRESS-CTO registry [14]. Technical and procedural success rates have not altered even if fewer retrograde procedures are being used. Along with operation duration, radiation, and contrast use, the rate of significant adverse cardiac events that occur in hospitals has also decreased [14]. This implies that without sacrificing technological advancement, contemporary CTO-PCI has grown safer and more effective. 

The present meta-analysis compares outcomes between antegrade and retrograde crossing techniques in CTO-PCI. The results of this meta-analysis showed that technical and procedural success were significantly better in patients who underwent antegrade CTO-PCI compared to their counterparts. Additionally, the risk of MACE, all-cause mortality, and myocardial infarction was also significantly lower in patients who underwent antegrade CTO-PCI compared to retrograde CTO-PCI. After analyzing 12 observational studies with 10,240 patients, Magaly et al. discovered that CTO lesions treated with an antegrade-only strategy had a greater success rate and a lower J-CTO score [26].

According to our research, the retrograde method increases the risk of myocardial infarction because of the prolonged occlusion of collateral channels by the microcatheters and retrograde wire [26], which has been linked to worse long-term outcomes and increased mortality. However, an antegrade-only strategy would not have been able to revascularize the majority of the CTOs included, and some of the issues linked to the retrograde approach would have arisen during antegrade crossing attempts. Furthermore, patients from the early 2000s were included in some of the studies in our study, which was at an early stage of the retrograde procedures' learning curve. A higher risk of restenosis is linked to the implantation of longer, smaller-caliber stents, which is typically the result of retrograde PCI in more difficult anatomical settings [27]. Furthermore, the retrograde approach's common utilization of dissection and re-entry procedures may raise the rate of restenosis [28-29]. 

Retrograde CTO-PCI procedures have become less common in modern practice due to concerns about the rate of problems associated with them. The usage of retrograde procedures decreased statistically significantly from 39% in 2012-2016 to 29% in 2017-2019, according to data from the PROGRESS-CTO registry [14]. Technical and procedural success rates have not altered, even if fewer retrograde procedures are being used. Along with operation duration, radiation, and contrast use, the rate of significant adverse cardiac events that occur in hospitals has also decreased [14]. This implies that, without sacrificing technological advancement, contemporary CTO-PCI has grown safer and more effective.

CTO-PCI has grown safer and more effective as a result of improved methods and tools. The use of retrograde methods has been a significant development in CTO-PCI. Rates of technical success associated with antegrade techniques (dissection reentry and wire escalation) have historically plateaued between 60% and 70% [30]. Modern rates of success among seasoned operators incorporating retrograde procedures consistently hover around 90%. Moreover, the intricacy of CTO-PCI lesions has progressively advanced. Retrograde techniques are regularly utilized for lesions with greater anatomical complexity and in patients burdened with multiple comorbidities, as shown by comprehensive data. In the field of CTO-PCI, continuous research aims to achieve technical success while managing the often observed, though slightly increased, risk of periprocedural complications [31]. 

As a rescue strategy after antegrade failure, observational research to date has pointed out no discernible differences in the frequency of problems with primary retrograde procedures versus secondary retrograde procedures [31]. The relevance of these results to practice is still up for debate. It is difficult to distinguish the role of retrograde procedures from increased-risk patients and the lesion anatomy treated in retrograde situations. Are more complex lesions and sicker patients inevitably associated with higher complications, or are retrograde procedures themselves the cause of increased complication rates? Understanding the relative contributions of patient and technique-specific factors can aid operators in providing a clear risk assessment and in determining the relative importance of primary retrograde strategies versus antegrade dissection reentry strategies when antegrade wiring is not practical, even though it is not a reason to always avoid a strategy or procedure.

There is still a tiny percentage of patients for whom all presently available procedures are ineffective, even after antegrade and retrograde approaches in CTO-PCI have been refined. It will take innovative approaches to treat these patients, especially if they are not candidates for surgery. The few CTOs that remain after all current methods have failed could be resolved by retrograde approaches aided by direct retrograde access from either an adjacent coronary vein or via video-assisted percutaneous pericardial access. In patients with significant comorbidity burdens and more complex lesions, retrograde procedures are still essential to the technical success of CTO-PCI. Appropriate retrograde conduit selection and CTO-PCI success rates will be significantly impacted by improvements in procedural safety through equipment iteration and adjunctive imaging utilization.

*Study Limitations* 

The present meta-analysis has certain limitations. First, only observational studies comparing antegrade and retrograde CTO-PCIs were conducted, which is attributed to selection bias. It was not possible to do additional research since the specifics of the crossing methods and collaterals utilized for the retrograde approach were not consistently disclosed in all of the included studies. The adjusted odds ratios were not provided by the included studies. Therefore, even though it was the best statistical technique, we were unable to validate our findings using the pooled adjusted RR. Therefore, in the future, more clinical trials will be required to compare these two techniques for developing clinical practice guidelines in order to improve patients' prognoses.

## Conclusions

This meta-analysis comparing antegrade and retrograde techniques in CTO-PCI reveals significant advantages for the antegrade approach. Antegrade CTO-PCI demonstrated higher technical and procedural success rates, along with lower risks of major adverse cardiac events, all-cause mortality, and myocardial infarction. However, the retrograde approach remains crucial for complex lesions and patients with multiple comorbidities. The declining use of retrograde procedures, coupled with improving success rates and safety profiles, indicates evolving practices in CTO-PCI. Future advancements in imaging and equipment may further enhance procedural safety and success rates. Despite limitations such as potential selection bias in observational studies, this analysis provides valuable insights for clinical decision-making in CTO-PCI.
